# All It Takes for Corruption in Health Systems to Triumph, Is Good People Who Do Nothing: Comment on "We Need to Talk About Corruption in Health Systems"

**DOI:** 10.15171/ijhpm.2019.53

**Published:** 2019-07-06

**Authors:** Saskia Mostert, Gertjan Kaspers

**Affiliations:** ^1^Department of Pediatrics, Amsterdam UMC, Amsterdam, The Netherlands.; ^2^Princess Máxima Center for Pediatric Oncology, Utrecht, The Netherlands.

**Keywords:** Corruption, Health Sector, Low and Middle-Income Countries

## Abstract

Numerous investigations demonstrate that the problem of corruption in the health sector is enormous and has grave negative consequences for patients. Nevertheless, the problem of corruption in health systems is far from eminent in the international health policy debate. Hutchinson, Balabanova, and McKee have identifed in their Editorial five reasons why the health policy community has been reluctant to talk about it: (1) Problem of defining corruption; (2) Some corrupt practices are actually ways of making dysfunctional systems work; (3) The serious challenges to researching corruption; (4) Concerns that focus on corruption is a form of victim blaming that ignores larger issues; and (5) Lack of evidence about what works to tackle it. In this commentary, we pay a closer and critical look at these five excuses for doing nothing. We conclude that the vast majority of the world population, being the poor in low and middle-income countries (LMICs) who disproportionately suffer from the problem of corruption in health systems, need good people with high moral and ethical principles who have the courage to disregard these five reasons. The poor need good people who understand that it is crucial to first acknowledge this problem, despite the obvious uncertainties involved, before you can change it. The poor therefore need good editors, good policy-makers, good managers, and good clinicians. We agree with the authors that we first need to talk about corruption. But above all, we need good people who are subsequently willing to walk the talk.

The editorial by Hutchinson et al^1^ starts with pointing out that some issues, which are wrong by any moral or ethical framework, are seldom discussed in public. This silence leads to a passive acceptance that nothing can be done to stop it. The authors subsequently state that the medical scientific and health policy community have their own dirty secret. It is corruption in health systems. The authors notice that our failure to confront this problem is all the more astonishing given that numerous investigations, for instance research performed by Transparency International, repeatedly confirm that the health sector is among the most corrupt in many nations worldwide. Corruption in health systems subverts the trust that supports efficient and fair medical care. And corruption costs lives. In the editorial the researchers reflect on the five reasons why it apparently is so difficult for the health policy community to initiate a debate on corruption in health systems: (1) Problem of defining corruption; (2) Some corrupt practices are actually ways of making dysfunctional systems work; (3) The serious challenges to researching corruption; (4) Concerns that focus on corruption is a form of victim blaming that ignores larger issues; and (5) Lack of evidence about what works to tackle it.^1^

In this commentary, we would like to start with emphasizing that a problem that is not acknowledged, cannot be changed. In order to address corruption in health systems, you first need to acknowledge its existence. And indeed we therefore fully agree with the authors that we must talk about this dirty and well-kept secret that adversely affects the health of the vast majority of the population worldwide.^2^ In fact, 80% of the global population lives in low and middle-income countries (LMICs) that are severely impacted by corruption. Within these countries, most people are from poor socio-economic backgrounds and disproportionately suffer from the problem of corruption in their health systems.^2^

Even the most prestigious medical scientific journals claim that they want to advance the science and art of medicine to ameliorate the public health by fostering a responsible and balanced discourse on significant issues that impact medicine, health care and health policy. So why are these medical scientific journals, that should guide policy-makers, managers, and clinicians, so reluctant to discuss the most important problem that hinders good medical care and public health in numerous countries worldwide?^1-3^ The editorial identifies five reasons not to discuss corruption within health systems.^1^ In this commentary, we would like to pay a closer look at these five excuses for doing nothing.

The first excuse is that it is difficult to define corruption.^1^ However, a very clear and simple definition has been provided by Transparency International: “abuse of entrusted power for private gain.”^3^ This definition covers all aspects of corruption within societies, including the health care sector. Corruption intentionally subdues public well-being to private welfare. Conduct of corruptors is characterized by dualism as it derives from both their profession and their self-interest. Corruption predominantly damages the poor as they can neither pay bribes nor afford private alternatives.^4-7^

The second excuse for doing nothing is that some corrupt practices are actually ways of making dysfunctional systems work.^1^ This misconception has very serious and even deadly consequences. Corruption has a major effect on the structure of health systems.^2^ By denying that this dysfunctional structure actually causes the problem, it cannot be rooted out. This misconception and denial has led to numerous invain efforts to improve the access and quality of medical care for patients in LMICs focusing on financing direct needs, like drugs or medical devices.^2,7^ The structure in which health systems operate is commonly disregarded. However, this dysfunctional structure sustains corruption and obstructs quality health care entry for poor people.^2,7^ The following key components of health system structures affected by corruption have been identified: “(*a*) Absent or failing monitoring systems for health budgets, personnel, and supplies; (*b*) No reward for good performance; (*c*) No punishment for misconduct; (*d*) Salaries for healthcare providers in public hospitals not in line with their educational background, skills, and training; and (*e*) Physician dual practices, absenteeism, and informal payments.”^2^ Governments of countries in which corruption is rife need to be pressured to address these key components of dysfunctional health systems in order to make a change.^2,3^

The third reason not to discuss the problem of corruption in health systems concerns the serious challenge to investigate corruption.^1^ This is a crucial point. Indeed, the current and rightful trend in medicine is that it should be evidence-based. However, the intrinsic nature of corruption implies that it involves secrecy and is therefore very much hidden.^2,4^ Consequently, the problem of corruption cannot be investigated as rigorously as other fields in medicine. Its investigation therefore will always be surrounded by more uncertainties than ideally desired. Unfortunately, this very aspect has discouraged many editors from medical scientific journals, who have the important responsibility to guide policy-makers, managers, and clinicians, from publishing important insights regarding the problem of corruption in health systems. Multiple times this was the reason why editors of so-called high-ranking journals decided not to publish our studies on corruption and hospital detention practices despite praising external peer reviews.^2^ An editorial decision to not publish and share crucial knowledge on corruption due to lack of empirical evidence, denies the striking reality that corruption paralyzes the provision of good medical care and public health.^1-3^ Corruption in medicine kills patients.^1,2^ If editors decide not to talk about corruption due to lack of evidence, they should realize that by doing nothing, they facilitate the triumph of corruption in health systems. If editors are silent, policy-makers, managers, and clinicians are not informed. Without information, they cannot make proper decisions and introduce policies that could tackle corruption and benefit the vast majority of the world population, being the poor in LMICs.^2^ Maybe it is therefore time to deal with the harsh reality and acknowledge that there are two categories of topics in medical science: those that can be fully evidence-based investigated and those that cannot. The latter studies, despite its obvious limitations, need to be shared with the health policy community, managers, clinicians and the general public in order to raise awareness about the problem of corruption in health systems and implement strategies that can actually save lives.^1,2^

The fourth excuse addresses concerns that focusing on corruption is a form of victim blaming that ignores larger issues.^1^ In this regard, two important questions need to be answered. First of all, who are the real victims? And secondly, what are the larger issues at stake? The answer to the first question is clear: the victims are predominantly the poor in LMICs whose access to proper medical care is denied because they cannot afford to pay bribes or private alternatives.^2,3^ But what are the larger issues? The authors refer to the unequal distribution of global resources.^1^ Focus on corruption does however not imply that this unequal distribution is ignored. It is in fact the opposite, focusing on the root causes of corruption acknowledges this large issue and may help to address it.^2^ Historically, high-income countries have profited from the rich natural resources in their former colonies, from its corrupt local leaders and from its poor subordinates. But also today government and business leaders from high-income countries bribe local leadership to secure contracts and impinge state decrees.^2,4,8-10^ These government and business deals foremost benefit the high-income countries, but also enrich local leaders, contaminate the environment, and pauperize the population.^2,4,8-10^ Politicians in high-income countries are silent about the dismal role their companies play in encouraging corruption in LMICs.^2,4,8-10^ And also the media in these high-income countries, including medical scientific journals, are unwilling to report the genuine magnitude of the problem of corruption in LMICs and how it is stimulated by high-income countries.^2,4,8-10^ Without realistic accounts, critical awareness and indispensable amendments for the poor population are precluded.^2,3^

The fifth excuse to do nothing is closely linked to the third: there is no research or empirical evidence yet how to effectively tackle corruption.^1^ Although this might be true in many LMICs, the passage of legislation and establishment of agencies to enforce the laws in Europe, Canada, and the United States has successfully evolved in civil and criminal investigations and convictions of government leaders and healthcare policy-makers. Unfortunately the latter, even when the evidence is conclusive, still do not receive the punishment they deserve in LMICs troubled by widespread corruption. This can partially be explained by the collectivist orientation in most of these LMICs. Preservation of social harmony and the culture of respect to those who hold higher positions in society, like government leaders, policy-makers and hospital managers, are given absolute priority and prevent that superiors can be criticised or hold accountable. When misconduct is not penalised, corruption in these settings cannot be tackled.^2,4^ This should however not stop editors, policy-makers, managers, and clinicians to acknowledge that the problem exists. Once the full extent and pervasiveness of the problem is acknowledged in medical science, policy-makers, managers, and clinicians can set up studies to investigate the effectiveness of intervention strategies. We would like to repeat here that you cannot change, what you do not acknowledge.^2,3^ To illustrate this, we will share some of our own pediatric oncology outreach program experiences with you.^2,11-15^ The survival of childhood cancer may be as high as 80% in high-income countries and is frequently less than 30% in LMICs. One of the most important, yet commonly unreported, root causes for this large gap in survival is the problem of corruption in health systems.^2,11-15^ A few examples of the effects of corruption on cancer care in LMICs^2^ are: (*a*) Inexperienced medical staff provide complex medical care without supervision because the experienced doctors, who receive full-time salaries to staff public hospitals, are working in their private practices; (*b*) Scarce drugs and medical devices are overpriced, substandard or counterfeit; (*c*) Medical equipment in public hospitals is intentionally disabled and repair overpriced and adjourned to wrongly refer patients to private health facilities; (*d*) Closed departments and facilities; (*e*) Long waiting lists; (*f*) Delayed diagnostics and late or interrupted cancer treatment; (*g*) The bad reputation of the public health sector stimulates the use of alternative treatment; (*h*) The postponed seeking of medical help leads to advanced and often no longer curable stages of disease at diagnosis.^2,12-15^ The ultimate outcome of corruption in health systems is unnecessary poor childhood cancer survial.^2,11-15^ Yet, the public health community does not see the urgency to acknowledge and address this dirty and well-kept secret. Maybe because the editors of medical scientific journals and global policy-makers have not witnessed themselves how innocent children are detained inside hospitals and corpses of patients are dumped in anonymous graves over unpaid medical bills.^2,12-15^ Maybe because they did not set up studies themselves to get the painful truth out.^2,12-15^ Maybe because they did not listen to the numerous accounts of the suffering families involved.^2,11-15^ Maybe for these reasons it is relatively easy for them to put these studies in the “too difficult” tray, as rightfully mentioned in the editorial of Hutchinson et al,^1^ with the excuse that there is not enough hard evidence to meet their high scientific standards. Maybe it is therefore that they choose to do nothing.

However, the vast majority of the world population does not need editors of medical scientific journals and global policy-makers who solely and rigorously focus on evidence-based medicine and thereby choose to look away and do nothing. The poor in LMICs need good and courageous people with high moral and ethical principles who realize that it is important to first acknowledge the problem of corruption in health systems, despite the obvious uncertainties involved, before you can change it. The poor need good editors, good policy-makers, good managers, and good clinicians. So yes, we fully agree with the authors that we first need to talk about corruption. But above all, we need good people who are subsequently willing to walk the talk.

## Ethical issues

Not applicable.

## Competing interests

Authors declare that they have no competing interests.

## Authors’ contributions

SM drafted the paper, which GK revised.

## Authors’ affiliations

^1^Department of Pediatrics, Amsterdam UMC, Amsterdam, The Netherlands. ^2^Princess Máxima Center for Pediatric Oncology, Utrecht, The Netherlands.
